# Database screening as a strategy to identify endogenous candidate metabolites to probe and assess mitochondrial drug toxicity

**DOI:** 10.1038/s41598-023-49443-0

**Published:** 2023-12-12

**Authors:** Mery Vet George De la Rosa, Dipali Patel, Marc R. McCann, Kathleen A. Stringer, Gus R. Rosania

**Affiliations:** 1 Department of Pharmaceutical Sciences, College of Pharmacy, University of Michigan, Ann Arbor, MI 48104 USA; 2 The NMR Metabolomics Laboratory, Department of Clinical Pharmacy, College of Pharmacy, University of Michigan, Ann Arbor, MI 48109 USA; 3 Division of Pulmonary and Critical Care Medicine, Department of Medicine, School of Medicine, University of Michigan, Ann Arbor, MI 48109 USA; 4 Weil Institute for Critical Care Research and Innovation, University of Michigan, Ann Arbor, MI 48109 USA

**Keywords:** Drug safety, Target identification

## Abstract

Adverse drug reactions (ADRs) are considered an inherent risk of medication use, and some ADRs have been associated with off-target drug interactions with mitochondria. Metabolites that reflect mitochondrial function may help identify patients at risk of mitochondrial toxicity. We employed a database strategy to identify candidate mitochondrial metabolites that could be clinically useful to identify individuals at increased risk of mitochondrial-related ADRs. This led to l-carnitine being identified as the candidate mitochondrial metabolite. l-carnitine, its acetylated metabolite, acetylcarnitine and other acylcarnitines are mitochondrial biomarkers used to detect inborn errors of metabolism. We hypothesized that changes in l-carnitine disposition, induced by a “challenge test” of intravenous l-carnitine, could identify mitochondrial-related ADRs by provoking variation in l-carnitine and/or acetylcarnitine blood levels. To test this hypothesis, we induced mitochondrial drug toxicity with clofazimine (CFZ) in a mouse model. Following CFZ treatment, mice received an l-carnitine “challenge test”. CFZ-induced changes in weight were consistent with previous work and reflect CFZ-induced catabolism. l-carnitine induced differences in whole blood acetylcarnitine concentrations in a manner that was dependent on CFZ treatment. This supports the usefulness of a database strategy for the discovery of candidate metabolite biomarkers of drug toxicity and substantiates the potential of the l-carnitine “challenge test” as a “probe” to identify drug-related toxicological manifestations.

## Introduction

Adverse drug reactions (ADRs) often result from drug effects unrelated to the drug’s primary mechanism of action. Even though ADRs are considered an inherent risk of medication use, they can have detrimental consequences to patients and pose a major burden on the United States healthcare system^1–3^. Most drugs are designed to directly activate or inhibit specific molecular targets involved in pathological processes, yet many ADRs have been found to be toxicological manifestations of off-target drug interactions affecting the structure and function of cellular organelles, such as mitochondria^4–6^. Healthy, fully functional mitochondria are vital for metabolic homeostasis because they are essential for both energy metabolism and the management of catabolic and anabolic processes; they also participate in a variety of signaling pathways^7–9^. Presently, there are no clinically utilized specific biomarkers of mitochondrial toxicity, and such testing is not required by the United States Food and Drug Administration (FDA) as part of the drug approval process^10,11^. This is the case despite that numerous investigations have shown that mitochondrial dysfunction is a major mechanism of drug-induced injury^11–13^.

Nevertheless, a number of studies have demonstrated gene-mitochondrial disease associations that have led to the development of early clinical diagnosis assays which are used for routine newborn testing to identify inborn errors in metabolism^14,15^. Existing databases of centralized information, such as Gene Expression Omnibus (GEO), Gene Ontology (GO), and Kyoto Encyclopedia of Genes and Genomes (KEGG), could be used to accelerate and improve the discovery of potential targets, biomarkers, and tracers that could aid in the assessment of mitochondrial-related ADRs^16–18^. The strategy of database screening has been used in various fields of study including to identify cancer-associated gene products and has resulted in the discovery of a prostate cancer-related gene, PAGE-1/GAGE-B^19^, a Ewing’s sarcoma-associated gene, XAGE-1^20^, and a number of differentially expressed transcripts in breast cancer^21^ and glioblastoma multiforme^22^. It has also been valuable in the identification of biomarkers for different diseases such as endometriosis^23^. However, these strategies have not been used to identify candidate metabolic biomarkers of mitochondrial-related ADRs.

To address this need, we developed a well-defined methodological approach to identify mitochondria-specific metabolites that could be clinically employed to probe and assess mitochondrial drug toxicity. We proceeded to identify a candidate metabolic biomarker that met all a priori criteria, followed by in vivo evaluation in a mouse model to corroborate the database screening algorithm and establish its feasibility for clinical use.

## Results

**Figure 1:**
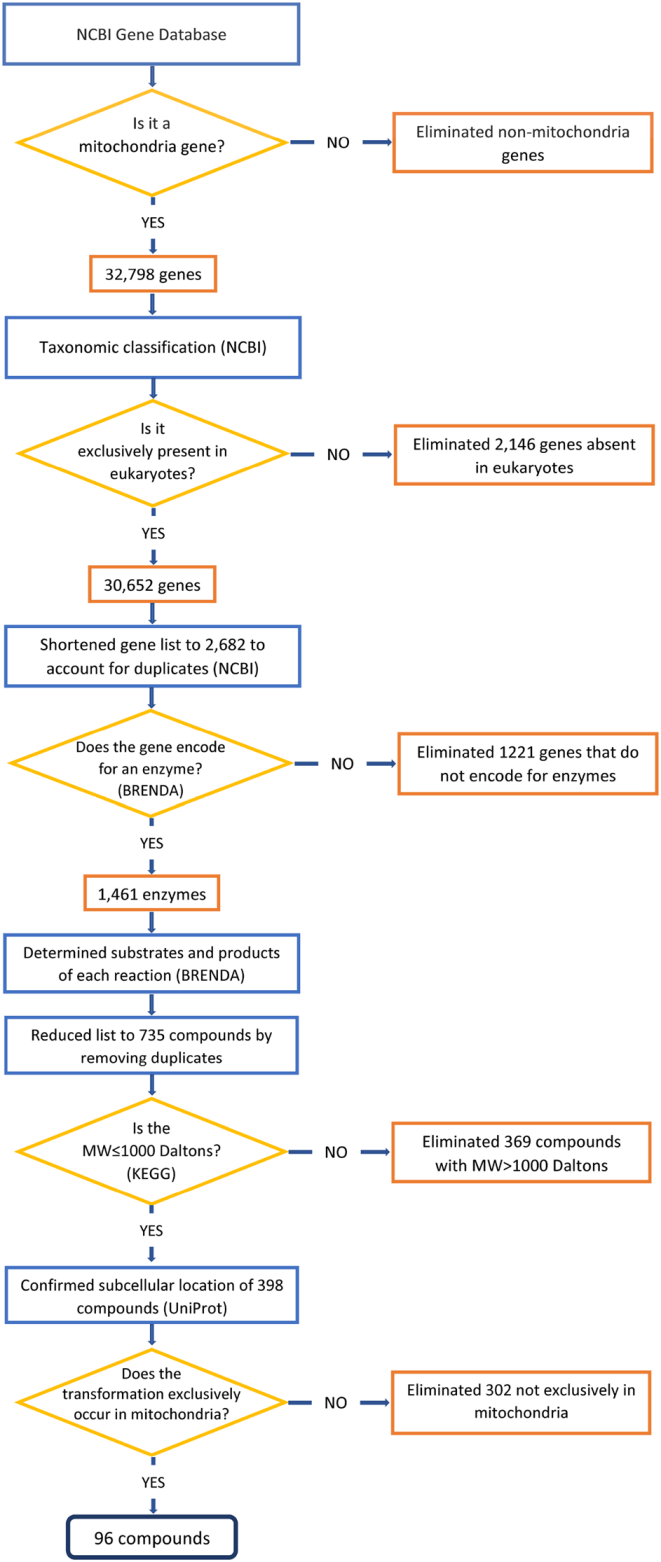
Methodology workflow diagram of the databases used to search for the candidate mitochondrial metabolites. The final 96 candidate metabolites were subjected to additional evaluation criteria which included assessment of physiochemical and pharmacokinetic properties and feasibility for clinical use. The workflow follows unified modeling language and includes the established a priori criteria used for each step. The rationale and the process for each step can be found in Supplementary Table S2 online.

A systematic algorithm based on rational, a priori criteria was developed for discovering mitochondrial-specific, candidate metabolites (Fig. 1 and Supplementary Table S1 online). Initially, 32,798 genes were identified by the National Center for Biotechnology Information's (NCBI) Gene database, 30,652 were categorized as eukaryotes genes, 1348 bacterial, 521 of archaea and 277 as virus (Supplementary Fig. S1 online). Only the 30,652 genes categorized as eukaryote were considered further. After screening for “eukaryote” mitochondrial genes, use of the GeneID reduced the list of relevant mitochondrial genes from 30,652 to 2682 which also accounted for duplicate entries (Supplementary Fig. S1 online). This step also permitted the building of a dataset with a gene symbol, gene description and gene synonyms for all similar genes that were present in the original list.

Use of the Braunschweig Enzyme Database (BRENDA) identified enzymes encoded by the remaining 2682 genes; 1461 enzymes were identified. The other 1221 genes were eliminated from further consideration since they were found to encode for protein structural components. The 1461 enzymes were combined into one data set that included the substrate and product of each enzymatic reaction. All duplicate compounds that were found to be substrates or products in multiple reactions were removed. This step resulted in 735 candidate compounds. We then used the Kyoto Encyclopedia of Genes and Genomes (KEGG) database (https://www.genome.jp/kegg/) to categorize the compounds by molecular weight (MW ≤ 1000 Daltons)^24,25^ which reduced the remaining 735 candidates to 398 (Fig. 1). Of the 398 remaining candidate compounds, the Universal Protein Resource (UNIPROT) database (https://www.uniprot.org/) confirmed that 96 were exclusively present in the mitochondria. The results from each step of the database screening methodology and data reduction steps are summarized in Fig. 1 and the rationale and process for each of the steps is summarized in Supplementary Table S2 online.

**Figure 2:**
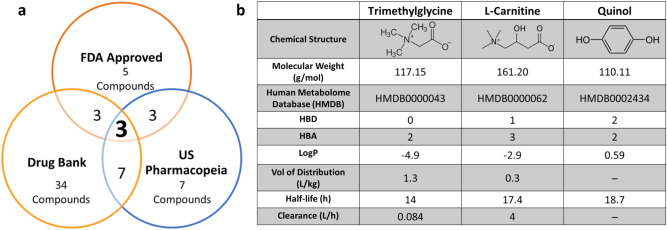
Final metabolite candidates following application of a database screening methodology. (**a**) Venn diagram shows the number of the final 96 compounds that were present in at least one of the three databases searched for physiochemical and pharmacokinetic properties: the FDA Orange Book (https://www.fda.gov/drugs/drug-approvals-and-databases/orange-book-data-files), Drug Bank (https://go.drugbank.com/) and the US Pharmacopeia (https://www.usp.org/). Only three compounds were present in all three databases. (**b**) Table of relevant physicochemical and pharmacokinetic properties of the three final candidate metabolites. HBD = Hydrogen Bond Donor; HBA = Hydrogen Bond Acceptor; LogP represents the logarithm (base 10) of the partition coefficient (P), the ratio of the compound's organic (oil)-to-aqueous phase concentrations; Vol = volume. Chemical structures in (**b**) were created in ChemDraw V20.0 (PerkinElmer Informatics, Waltham, MA USA).

Of the final 96 compounds, seven were found in the US Pharmacopeia, 34 compounds were in Drug Bank and only five were found to be FDA approved for human use. Three of these compounds were in all three databases (Fig. 2a), which made them finalist candidates: trimethylglycine (betaine; HMDB0000043), l-carnitine (HMDB0000062), and quinol (hydroquinone; HMDB0002434; Fig. 2). Figure 2b shows the physiochemical and pharmacokinetic properties of the three final compounds.

**Figure 3:**
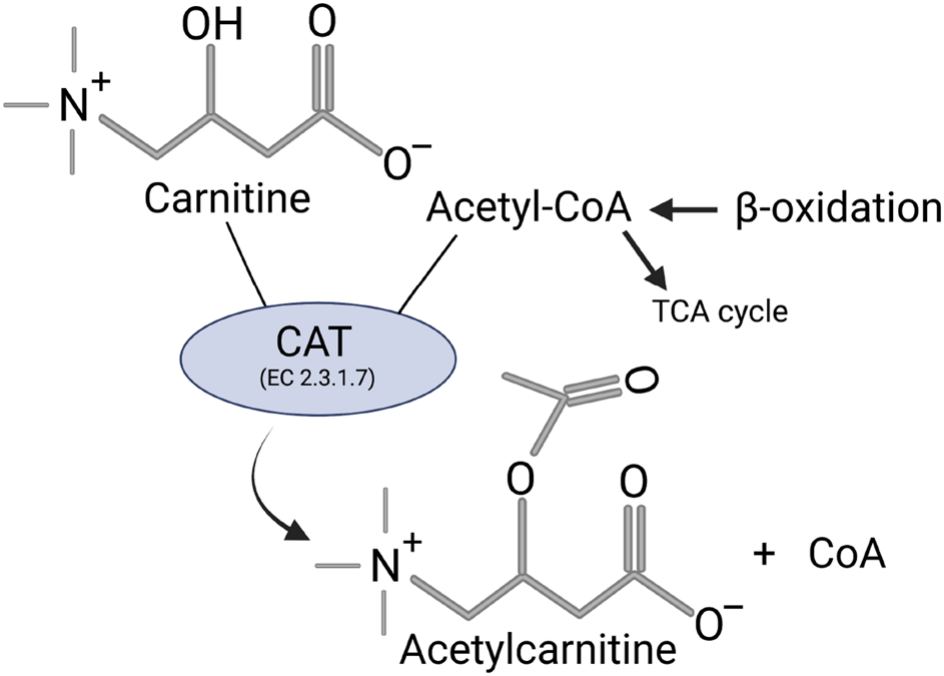
Generation of acetylcarnitine from l-carnitine occurs via carnitine acetyltransferase (CAT; EC 2.3.1.7). In mitochondria, the production of acetylcarnitine is catalyzed by CAT from l-carnitine and acetyl-CoA, a product of beta-oxidation and substrate for the tricyclic acid (TCA) cycle. In the setting of a surplus of l-carnitine as would occur from an l-carnitine “challenge test”, and a reduced supply of acetyl-CoA secondary to clofazimine-induced metabolic stress (e.g., catabolism), the production of acetylcarnitine may be reduced compared to a non-catabolic state. Figure created by BioRender.com.

A comprehensive evaluation of published research was used to rank the relevance of the final selected compounds as biomarkers based on their known association with ADR’s, mitochondrial function, and known animal and human physiological blood concentrations (Supplementary Table S3 online). Accordingly, l-carnitine was selected as the most clinically feasible candidate as it is available as an FDA approved formulation for intravenous injection (Carnitor^®^, Leadiant Biosciences, Gaithersburg, MD; USA). l-carnitine is converted to acetylcarnitine via carnitine acetyltransferase (EC 2.3.1.7) in mitochondria (Fig. 3), acetylcarnitine is also the most abundant acylcarnitine, which made it the metabolite of choice for further testing as a metabolic tracer of mitochondrial metabolism.

### In vivo l-carnitine challenge

**Figure 4:**
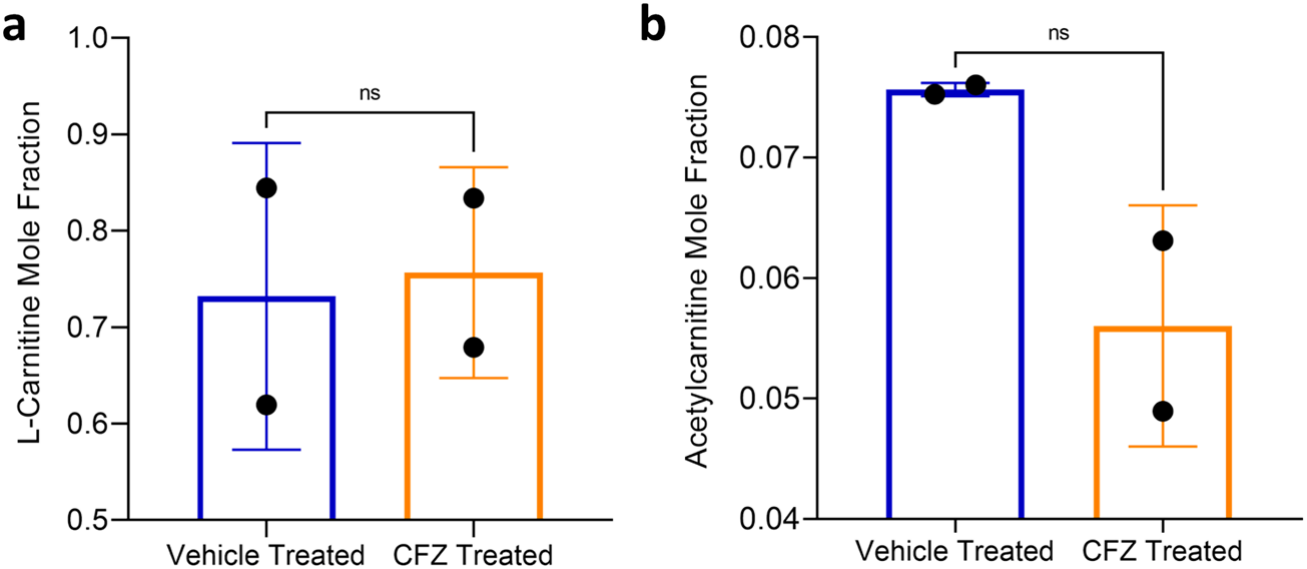
The mole fraction of l-carnitine and acetylcarnitine in the urine 24 h after a bolus IV l-carnitine injection were not changed by clofazimine (CFZ) treatment. (**a**) The urine mole fraction of l-carnitine in CFZ (orange) and vehicle treated (blue) mice was 0.73 and 0.76, respectively (p = 0.87). (**b**) The urine mass ratio of acetylcarnitine in CFZ treated and vehicle treated mice was 0.6 and 0.76, respectively (p = 0.11). Data are the mean (SD) of two metabolic cages each housing 5 mice/group. Mole fraction of l-carnitine and acetylcarnitine was calculated by dividing the amount of l-carnitine or acetylcarnitine recovered in urine collected over 24 h following the administration of the l-carnitine challenge dose.

**Figure 5:**
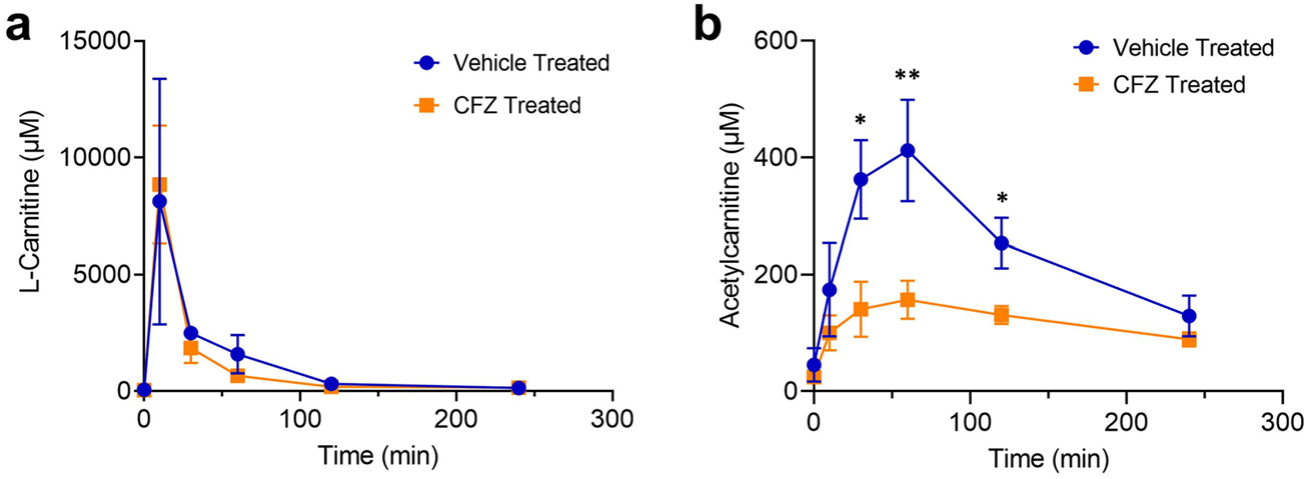
Whole blood l-carnitine and acetylcarnitine concentrations (µM) in clofazimine (CFZ) and vehicle treated mice following the l-carnitine “challenge test” (1000 mg/kg). (**a**) Following tail vein injection of l-carnitine, vehicle and CFZ treated mice had similar l-carnitine levels in whole blood. (**b**) Following tail vein injection, CFZ treated mice had lower acetylcarnitine levels at 30 min (*p = 0.0138), 60 min (**p = 0.0024), and 120 min (*p = 0.0465) compared with vehicle treated mice. Pre-treatment levels and at 10 min (p = 0.379) the difference was not significant. Data are the mean (SD) of 10 mice/group.

l-carnitine was evaluated to assess the validity of our database strategy and its feasibility for clinical use as a marker of mitochondrial-related ADR using CFZ-treated mice as a model. The results from these experiments are depicted in Figs. 4 and 5 and Supplementary Figs. S2–S4 online.

In urine, the amount of both l-carnitine and its primary metabolite, acetylcarnitine (HMDB0000201), was not different between the two treatment groups (Fig. 3).

Following tail vein injection of l-carnitine (1000 mg/kg), vehicle and CFZ treated mice had similar l-carnitine levels in whole blood (Fig. 5a). However, the mean (SD) whole blood acetylcarnitine concentrations were lower in CFZ treated mice at 30 min (*p = 0.0138), 60 min (**p = 0.0024), and 120 min (*p = 0.0465) compared with vehicle treated mice. Pre-treatment and levels at 10 min (p = 0.379) were not different (Fig. 5b).

## Discussion

As reported herein, use of a database screening and process of elimination strategy, led to the identification of 96 candidate mitochondrial metabolites that could be measured in blood to provide useful readouts of drug-induced changes in mitochondrial function. Further selection based on clinical relevance and in vivo evaluation revealed l-carnitine as a highly feasible candidate for clinical use based on a priori criteria. Measured changes of acetylcarnitine levels following an intravenous injection of l-carnitine in animals exposed to a “mitochondriotoxic” drug corroborated the usefulness of the database approach for strategically identifying those endogenous metabolites that could serve as candidate biomarkers for detecting changes in metabolism induced by drugs that target a specific organelle. Of noteworthy significance, the existing information that was publicly available in databases allowed for a systematic process of elimination, which led to a concise list of clinically feasible human metabolite candidates for use as functional tracers of mitochondrial metabolic health.

**Figure 6:**
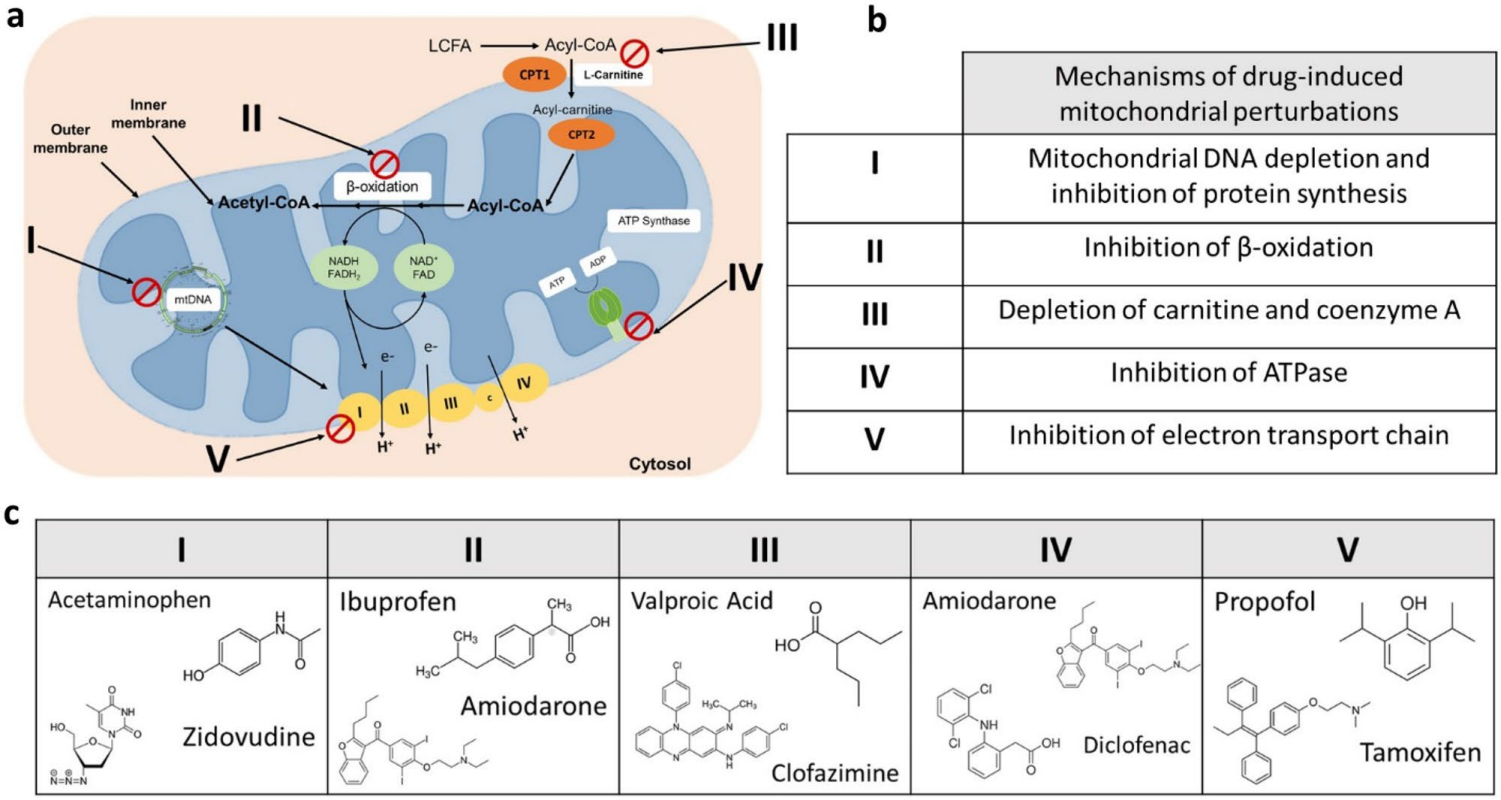
Mechanisms and pathways in mitochondria that are adversely affected by drugs. (**a**–**c**) I. mtDNA damage or depletion: damage to mtDNA by drugs (e.g., high dose acetaminophen^44^, zidovudine) impairs mtDNA replication and thus mitochondrial protein synthesis. (**a**–**c**) II. Inhibition of beta-oxidation of fatty acids: drugs like ibuprofen and amiodarone, inhibit either the enzymes involved in beta-oxidation or the ETC resulting in accumulation of fatty acids due to impaired beta oxidation. (**a**–**c**) III. Depletion of carnitine and CoA: the generation of certain CoA and/or l-carnitine esters by drugs like valproic acid, decreases the levels of these, hindering the mechanism of β-oxidation. (**a**–**c**) IV. Inhibition of ATPase: drugs like amiodarone and NSAIDs like diclofenac can cause oxidative phosphorylation uncoupling, causing a reduction in ATP production. (**a**–**c**) V. Electron transport chain (ETC) inhibition: inhibition of respiratory chain enzyme complex activities by drugs (e.g., propofol, tamoxifen) blocks electron transfer along the ETC. Inhibition of the mitochondrial ETC induces cell death through the generation of reactive oxygen species. Figure 5a was created with BioRender.com, all chemical structures in Fig. 5c were created in ChemDraw V20.0 (PerkinElmer Informatics).

Mitochondria possess diverse structural components and functional features which can be targeted by a compound and lead to toxicity^26,27^. These interactions can be caused by different types of drugs and occur in different sites and pathways (Fig. 6). These targets include inhibition of mitochondrial DNA (mtDNA) transcription of electron transport chain (ETC) complexes and enzymes required for any of the steps of glycolysis and β-oxidation, the depletion of l-carnitine and Coenzyme A (CoA), uncoupling of oxidative phosphorylation, and interference with one or more of the complexes in the respiratory chain, affecting the ETC. Early signals of many of these processes are difficult to detect in the blood.

While assessment of ADR-associated mitochondrial dysfunction is not currently part of the drug approval process, a blood assay that could be used to report drug-induced changes in mitochondrial function could be valuable. Mitochondria contain their own genome which can affect the probability of ADRs but the mitochondrial genome is not easily accessible in the clinical situation^27,28^. Rather, a circulating metabolite like l-carnitine, that could reflect the underlying cellular and molecular mechanisms that predisposes individuals to unintended perturbation of mitochondrial metabolic function by a medication, would be most clinically achievable and feasible. To this end, the algorithm developed herein could be clinically used to probe and assess a range of different adverse drug effects on the metabolic status of other organelles in addition to mitochondria (Fig. 1 and Supplementary Table S2 online).

The identified three final compounds were all mitochondrial metabolites. To pinpoint the most ideal candidate, we assessed information about the pharmacokinetic and physiochemical properties from different databases (Fig. 2b). l-carnitine is FDA approved for the treatment of carnitine deficiency and is available as an intravenous (IV) formulation (Carnitor^®^, Leadiant Biosciences, Gaithersburg, MD USA). Trimethylglycine, also known as betaine, is available as an FDA approved oral formulation (CYSTADANE^®^, Recordati Rare Diseases Inc, Lebanon, NJ, USA) that is used for the treatment of homocystinuria to decrease high homocysteine blood levels; currently, there is no IV formulation. As such, trimethylglycine was removed as a candidate. Quinol (hydroquinone) is an aromatic organic phenol which participates in the mitochondria Q cycle. This is the process by which the electrons are transferred from ubiquinol to cytochrome c, which results in the net movement of protons across the inner mitochondrial membrane^29^. Even though quinol has a critical role in the electron transport chain, as a therapeutic, it is used in combination with fluocinolone and tretinoin as an FDA-approved topical treatment for melasma; it is not available in a formulation for injection. In terms of the in vivo assay system that we chose to test the usefulness of l-carnitine, CFZ is an FDA-approved, weakly basic, red-pigmented, phenazine antibiotic that is included in the WHO List of Essential Medications as part of the standard treatment for leprosy^30,31^. It is highly lipophilic and is characterized by an unusually long elimination half-life (up to 70 days), which is associated with extensive accumulation of the drug in the body^31,32^. While CFZ is well tolerated, in addition to its adverse impact on mitochondrial function^32^, it imposes considerable metabolic stress on the host secondary to induction of a catabolic state^33–36^. Following 8-weeks of CFZ treatment we subjected mice to the l-carnitine challenge test (1000 mg/kg given as a single IV injection). Although we did not detect a CFZ-induced change in whole blood concentrations of l-carnitine, the-l-carnitine challenge test induced differences in whole blood acetylcarnitine concentrations in CFZ-treated mice. Specifically, in CFZ treated mice, acetylcarnitine concentrations were significantly lower when compared to vehicle treated mice (Fig. 5b). Typically, high dose l-carnitine supplementation leads to a proportional increase in blood levels of both l-carnitine and acetylcarnitine, the primary acetylated form of l-carnitine^37,38^. The function of this shuttle and l-carnitine are essential for the transport of long-chain fatty acids into the mitochondria. In normal, healthy individuals, skeletal muscle stores 97% of total body l-carnitine, while l-carnitine in blood accounts for only 0.1% of total body l-carnitine^39^. In the tissue compartment (including skeletal muscle) the generation of acetylcarnitine relies on the availability of two substrates-l-carnitine and acetyl-CoA (Fig. 3). The enzyme, CAT (EC 2.3.1.7), converts them to acetylcarnitine and CoA. A possible explanation for the decline in acetylcarnitine production in response to l-carnitine supplementation in CFZ-treated mice is a reduction in the availability of acetyl-CoA for acetylcarnitine generation secondary to CFZ-induced catabolism. High energy demand leads to consumption of acetyl-CoA leaving less reserve available for the production of acetylcarnitine^40^. This finding highlights the importance of measuring acetylcarnitine. A number of studies have relied on the measurement of acetylcarnitine and acylcarnitines as markers for different disease states and drug-induced ADRs^41^. The differences induced in acetylcarnitine blood levels by the l-carnitine “challenge test” in CFZ versus vehicle treated mice point to the feasibility of its use as a “probe” to identify drug-related mitochondrial and medication-induced toxicological manifestations.

We acknowledge that there are limitations to this study. First, we acknowledge that a database search methodology (which was conducted ~ 2 years ago) may not yield the exact same results today, in part, because the content of databases and website formats change over time. To combat this limitation, we have made the resulting data files publicly available. We also recognize that clofazimine is only one of many drugs that can affect mitochondrial function through a variety of different mechanisms. We acknowledge that the interaction between other mitochondriotoxic drugs and l-carnitine/acetylcarnitine transport, metabolism and disposition pathways could be very different from those that occur during CFZ treatment. This study was also limited to mice of the same genetic background. It therefore does not fully account for all the variation in l-carnitine and acetylcarnitine transport, metabolism and disposition that may characterize an entire population with different genetics, ages, and exposures to different environmental conditions that might lead to a more varied response to either CFZ or to an l-carnitine challenge test.

Despite these limitations, our prospective study identified functional biomarkers of mitochondrial toxicity that could be used to stratify individuals at increased ADR risk^42^. The findings described here serve as a starting point in the development of a test to probe the relationship between baseline metabolic stress and drug-related mitochondrial toxicity that could help determine which patients may be at risk. To further refine these measurements, a more detailed assessment of the distribution of l-carnitine and acetylcarnitine in response to the l-carnitine challenge test, could also be elaborated using an isotope labeled l-carnitine analog. Such a metabolic tracer could yield additional information about drug-induced changes to endogenous l-carnitine and acetylcarnitine stores, transport, and utilization.

## Methods

### Database screening methodology

The study was carried out following an a priori algorithm (Fig. 1) which was specifically designed to identify mitochondrial-related metabolites that could be clinically employed to probe and assess mitochondrial drug toxicity and direct the development of an in vivo and clinically-relevant “challenge test”. A set of criteria was established for each step of the workflow and each database that was screened (Supplementary Table S3 online); candidate metabolites were included or excluded from further consideration based on these criteria. For the methodology, we used four different databases in sequential order for the initial portion of the analysis: The National Center for Biotechnology Information’s (NCBI) Gene database (https://www.ncbi.nlm.nih.gov/gene), The Braunschweig Enzyme Database (BRENDA) (https://www.brenda-enzymes.org/), the Kyoto Encyclopedia of Genes and Genomes (KEGG) database (https://www.genome.jp/kegg/), and the Universal Protein Resource (UNIPROT) database (https://www.uniprot.org/). The resulting candidates were then subjected to evaluation by three criteria that were assessed using; Drug Bank (https://go.drugbank.com/), the FDA Orange Book (https://www.accessdata.fda.gov/scripts/cder/ob/index.cfm), and the United States Pharmacopeia (USP; https://www.usp.org/). The rationale for the use of each database, the sequence of evaluation and the inclusion/exclusion criteria for each candidate are outlined in Supplementary Table S2 online.

### In vivo l-carnitine challenge experiment

The main candidate metabolite identified by our database strategy was evaluated using a mouse model of mitochondrial drug toxicity. The animal protocol was approved by the University of Michigan’s Institutional Animal Care and Use Committee (protocol number PRO00009404) and animal care was provided in accordance with the NIH Guide for the Care and Use of Laboratory Animals. We also complied with the ARRIVE guidelines^43^.

**Figure 7:**
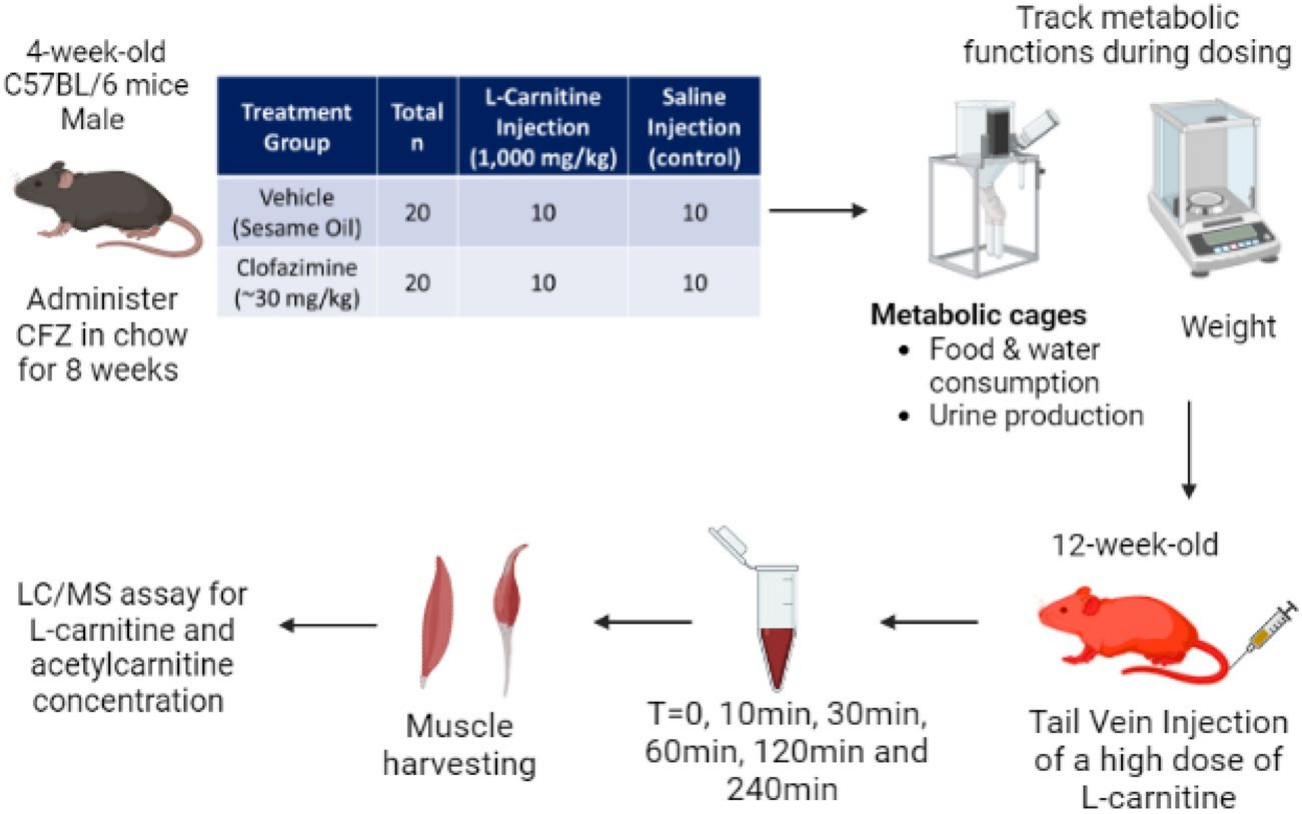
In vivo l-carnitine challenge test methodology and treatment groups**.** Figure was created with BioRender.com.

An l-carnitine “challenge” was used to provoke drug-related mitochondrial toxicological manifestations. (Fig. 7). Male C57BL/6 mice were treated with CFZ, an FDA approved medication known to cause mitochondrial dysfunction^36^, by its addition to chow for 8-weeks (~ 40 mg/kg) as previously described^36^. Following CFZ treatment, mice were injected with a high dose (1000 mg/kg) of l-carnitine, referred to as the “challenge test”. Metabolic functions were tracked, including weight, food and water consumption, and urine production. The amount of l-carnitine and acetylcarnitine in urine is shown in mole fraction. These values were calculated by dividing the amount of l-carnitine or acetylcarnitine recovered after 24 h in urine by the starting dose of the l-carnitine challenge injected. Pooled urine samples (5 mice/per sample) were collected at 24 h using metabolic cages (Techniplast^®^). Whole bloodsamples were collected via the saphenous vein (BL, 10 min, 30 min, 60 min, 120 min) and the retro-orbital plexus by removal of the eye while under anesthesia (inhaled isoflurane) for the terminal timepoint (240 min) then flash frozen in liquid nitrogen. At the study termination, mice were euthanized according to IACUC guidelines (Policy on Human Care and Use of Laboratory Animals Approved Animal Welfare Assurance Number, D16–00072 (A3114–01)). Sodium-heparin preserved whole blood and centrifuge-clarified urine samples were stored (− 80 °C) until the time of assay. l-carnitine and acetylcarnitine concentrations were measured using a quantitative liquid chromatography–mass spectrometry (LC/MS) assay.

### Quantification of l-carnitine and acetylcarnitine in blood and urine

Briefly, to determine l-carnitine and acetylcarnitine concentrations in mouse blood and urine, water (490 μL) was added into 10 μL blood or urine. Internal standard solution (5 μL; l-carnitine-13C, D3 and acetylcarnitine-D3, 5 μg/mL in acetonitrile, Thermo Fisher Scientific) was added to the diluted blood or urine samples and mixed for 30 min at room temperature. Finally, 150 μL of acetonitrile was added to precipitate macromolecules. The mixture was vortexed for 10 min and centrifuged (3000*g* for 10 min at 4 °C). The supernatant was transferred to the autosampler vials for LC–MS/MS analysis. Additional details about the assay can be found in the supplement.

To construct calibration curves for l-carnitine and acetylcarnitine, 5 μL internal standard solution was added into 100 μL of 12 nonzero mixed standards, which were prepared in water, and mixed for 30 min at room temperature. Then, 150 μL of Acetonitrile was added into 50 μL of the mixture. After vortexed and centrifuged with the conditions for samples, the standard solutions were injected to LC–MS/MS. By plotting the peak area ratio of l-carnitine or acetylcarnitine to the internal standard versus the sample concentration. The concentration range evaluated was from 1 to 5000 ng/ml. Quality control solutions were prepared from separate weighted powder to the concentration of 10, 250, 2500 ng/ml in water. Then, quality control samples were obtained from mixing of a diluted blood sample with above solution at 1:1 (*v*/*v*). Quality control samples were run before, in the middle and after the samples to evaluate the accuracy and intra-batch precision of the developed method.

### Data processing and statistical analysis

GraphPad Prism Version 9.3.1 (GraphPad Software, San Diego, CA, USA) was used for all statistical analysis. All data are presented as mean ± SD. Significance between different treatment groups, vehicle treated vs CFZ treated, was assessed by two-tailed unpaired Student’s t-test for the urine mole fraction, weight, food consumption, water consumption, urine volume and muscle mass. Significance between different treatment groups in whole blood l-carnitine and acetylcarnitine concentrations over time was assessed by a mixed-effects model with a Šidák multiple comparison correction. Statistical difference was considered at p < 0.05.

## Conclusions

This study, using a database search strategy, aimed to identify candidate mitochondrial metabolites that could be clinically useful to identify individuals at increased risk of mitochondrial-related ADRs. Mitochondrial impairment by drugs may involve interference with many different pathways and mechanisms, all of which can lead to unexpected ADRs. Clinical use of an l-carnitine challenge test with subsequent measurement of mitochondrial metabolites like acetylcarnitine, could be an important early step to identify occult medication-induced mitochondrial toxicity.

### Supplementary Information

Supplementary Information.

## Data Availability

The datasets generated and/or analysed during the current study are available in the University of Michigan’s Deep Blue data repository, 10.7302/gnx4-gs93.

## References

[1] Gyllensten H (2013). Cost of illness of patient-reported adverse drug events: A population-based cross-sectional survey. BMJ Open.

[2] Sultana J, Cutroneo P, Trifirò G (2013). Clinical and economic burden of adverse drug reactions. J. Pharmacol. Pharmacother..

[3] Lundkvist J, Jönsson B (2004). Pharmacoeconomics of adverse drug reactions. Fundam. Clin. Pharmacol..

[4] Garon SL (2017). Pharmacogenomics of off-target adverse drug reactions. Br. J. Clin. Pharmacol..

[5] Meyer JN, Hartman JH, Mello DF (2018). Mitochondrial toxicity. Toxicol. Sci..

[6] Young MJ (2017). Off-target effects of drugs that disrupt human mitochondrial DNA maintenance. Front. Mol. Biosci..

[7] Friedman JR, Nunnari J (2014). Mitochondrial form and function. Nature.

[8] Hoitzing H, Johnston IG, Jones NS (2015). What is the function of mitochondrial networks? A theoretical assessment of hypotheses and proposal for future research. BioEssays.

[9] Herst PM, Rowe MR, Carson GM, Berridge MV (2017). Functional mitochondria in health and disease. Front. Endocrinol..

[10] Scatena R (2011). Mitochondria and drugs. Adv. Mitochondrial Med..

[11] Dykens JA, Will Y (2007). The significance of mitochondrial toxicity testing in drug development. Drug Discov. Today.

[12] Nadanaciva S, Will Y (2009). Current concepts in drug-induced mitochondrial toxicity. Curr. Protoc. Toxicol..

[13] Wallace KB, Starkov AA (2000). Mitochondrial targets of drug toxicity. Annu. Rev. Pharmacol. Toxicol..

[14] Wang W (2021). The mining and construction of a knowledge base for gene-disease association in mitochondrial diseases. Sci. Rep..

[15] Shen L (2016). MSeqDR: A centralized knowledge repository and bioinformatics web resource to facilitate genomic investigations in mitochondrial disease. Hum. Mutat..

[16] Smith AC, Robinson AJ (2019). Mitominer v4.0: An updated database of mitochondrial localization evidence, phenotypes and diseases. Nucleic Acids Res..

[17] Apweiler R (2004). UniProt: The universal protein knowledgebase. Nucleic Acids Res..

[18] Schoch CL (2020). NCBI taxonomy: A comprehensive update on curation, resources and tools. Database.

[19] Brinkmann U (1998). PAGE-1, an X chromosome-linked GAGE-like gene that is expressed in normal and neoplastic prostate, testis, and uterus. Proc. Natl. Acad. Sci. USA.

[20] Liu XF (2000). XAGE-1, a new gene that is frequently expressed in Ewing’s sarcoma. Cancer Res..

[21] Schmitt AO (1999). Exhaustive mining of EST libraries for genes differentially expressed in normal and tumour tissues. Nucleic Acids Res..

[22] Loging WT (2000). Identifying potential tumor markers and antigens by database mining and rapid expression screening [5]. Genome Res..

[23] Lu Z, Gao Y (2021). Screening differentially expressed genes between endometriosis and ovarian cancer to find new biomarkers for endometriosis. Ann. Med..

[24] Hadacek F, Bachmann G (2015). Low-molecular-weight metabolite systems chemistry. Front. Environ. Sci..

[25] Maeda H, Matsumoto T, Konno T, Iwai K, Ueda M (1984). Tailor-making of protein drugs by polymer conjugation for tumor targeting: A brief review on smancs. J. Protein Chem..

[26] Vuda M, Kamath A (2016). Drug induced mitochondrial dysfunction: Mechanisms and adverse clinical consequences. Mitochondrion.

[27] Boelsterli UA, Lim PLK (2007). Mitochondrial abnormalities—A link to idiosyncratic drug hepatotoxicity?. Toxicol. Appl. Pharmacol..

[28] Penman SL, Carter AS, Chadwick AE (2020). Investigating the importance of individual mitochondrial genotype in susceptibility to drug-induced toxicity. Biochem. Soc. Trans..

[29] Li Q, Kang C (2020). Mechanisms of action for small molecules revealed by structural biology in drug discovery. Int. J. Mol. Sci..

[30] Cholo MC, Steel HC, Fourie PB, Germishuizen WA, Anderson R (2012). Clofazimine: Current status and future prospects. J. Antimicrob. Chemother..

[31] Murashov MD (2018). Synthesis and characterization of a biomimetic formulation of clofazimine hydrochloride microcrystals for parenteral administration. Pharmaceutics.

[32] Baik J, Rosania GR (2011). Molecular imaging of intracellular drug-membrane aggregate formation. Mol. Pharm..

[33] Baik J, Stringer KA, Mane G, Rosania GR (2013). Multiscale distribution and bioaccumulation analysis of clofazimine reveals a massive immune system-mediated xenobiotic sequestration response. Antimicrob. Agents Chemother..

[34] Yoon GS (2015). Phagocytosed clofazimine biocrystals can modulate innate immune signaling by inhibiting TNFα and boosting IL-1RA secretion. Mol. Pharm..

[35] Hastings R, Jacobson R, Trautman J (1976). Long-term clinical toxicity studies with clofazimine (B663) in leprosy. Int. J. Lepr. Other Mycobact. Dis..

[36] Trexel J (2017). Macrophage-mediated clofazimine sequestration is accompanied by a shift in host energy metabolism. J. Pharm. Sci..

[37] Ferreira GC, McKenna MC (2017). l-Carnitine and acetyl-l-carnitine roles and neuroprotection in developing brain. Neurochem. Res..

[38] Jennaro TS (2020). Using l-carnitine as a pharmacologic probe of the interpatient and metabolic variability of sepsis. Pharmacother. J. Hum. Pharmacol. Drug Ther..

[39] Rasmussen J (2014). Carnitine levels in skeletal muscle, blood, and urine in patients with primary carnitine deficiency during intermission of l-carnitine supplementation. JIMD Rep..

[40] Shi L, Tu BP (2015). Acetyl-CoA and the regulation of metabolism: Mechanisms and consequences. Curr. Opin. Cell Biol..

[41] McCann MR, George De la Rosa MV, Rosania GR, Stringer KA (2021). l-Carnitine and acylcarnitines: Mitochondrial biomarkers for precision medicine. Metabolites.

[42] Nadanaciva S, Will Y (2011). New insights in drug-induced mitochondrial toxicity. Curr. Pharm. Des..

[43] du Sert NP (2020). Reporting animal research: Explanation and elaboration for the arrive guidelines 2.0. PLoS Biol..

[44] Cover C (2005). Peroxynitrite-induced mitochondrial and endonuclease-mediated nuclear DNA damage in acetaminophen hepatotoxicity. J. Pharmacol. Exp. Ther..

