# Supplementary material for: Database screening as a strategy to identify endogenous candidate metabolites to probe and assess mitochondrial drug toxicity
Source: Sci Rep. 2023 Dec 12;13:22013. doi: 10.1038/s41598-023-49443-0 (PMC10716408; doi:10.1038/s41598-023-49443-0)
Supplement: Supplementary file 1 — Supplementary Information. [file 41598_2023_49443_MOESM1_ESM.docx]

*Supplementary Information*

Database Screening as a Strategy to Identify Endogenous Candidate Metabolites to Probe and Assess Mitochondrial Drug Toxicity

*Mery Vet George De la Rosa,^1^ Dipali Patel,^1^ Marc R. McCann,^2^ Kathleen A. Stringer,^2,3,4,^ and Gus R. Rosania^1*^*

^1^Department of Pharmaceutical Sciences, College of Pharmacy, University of Michigan, Ann Arbor, MI, 48104, USA.

^2^The NMR Metabolomics Laboratory, Department of Clinical Pharmacy, College of Pharmacy, University of Michigan, Ann Arbor, MI 48109, USA.

^3^Division of Pulmonary and Critical Care Medicine, Department of Medicine, School of Medicine, University of Michigan, Ann Arbor, MI 48109, USA

^4^Weil Institute for Critical Care Research and Innovation, University of Michigan,

Ann Arbor, MI 48109, USA

***** Correspondence: grosania@umich.edu; Tel.: +1-734-763-1032

**Results**

Application of a rigorous database screening approach with established a priori criteria for each step of the workflow methodology, yielded 96 mitochondrial candidate metabolites (**Supplementary Table S1**).

**Supplementary Table S1.** Candidate Mitochondrial Metabolites Identified from Database Screening

| **Metabolite** | **HMDB ID*** |
| --- | --- |
| (20R,22R)-20,22-dihydroxy-cholesterol | N/A |
| (2E)-2-enoyl-CoA | N/A |
| (2E,4E)-2,4-dienoyl-CoA | HMDB0301033 |
| (2E,6E)-farnesyl diphosphate | HMDB0000961 |
| (3R)-3-hydroxy-2-oxo-4-phosphooxybutanoate | HMDB0006801 |
| (3S)-3-hydroxyacyl-CoA | N/A |
| (6S)-6beta-hydroxy-1,4,5,6-tetrahydronicotinamide-adenine dinucleotide phosphate | N/A |
| (E)-but-2-enoyl-CoA | HMDB0002009 |
| (S)-1-pyrroline-5-carboxylate | HMDB0001301 |
| [(1->3)-beta-D-glucosyl] n | N/A |
| 1,2-diacyl-3-O-(beta-D-galactosyl)-sn-glycerol | N/A |
| 1,2-diacyl-3-O-[alpha-D-galactosyl-(1->6)-beta-D-galactosyl]-sn-glycerol | N/A |
| 2,3-dehydroacyl-CoA | N/A |
| 2-acetolactate | HMDB0006833 |
| 2-dehydropantoate | HMDB0304068 |
| 2'-deoxyribonucleoside 5'-triphosphate | N/A |
| 2-oxobutanoate | HMDB0000005 |
| 3-(methylsulfanyl)-L-aspartate89- [ribosomal protein S12] | N/A |
| 3-methylcrotonoyl-CoA | HMDB0001493 |
| 3-methylglutaconyl-CoA | HMDB0001057 |
| 3-oxo acid | N/A |
| 3-oxoacyl-CoA | HMDBP09209 |
| 3-phosphooxypyruvate | HMDB0001024 |
| 4 beta-D-galactopyranose | HMDB0246552 |
| 4-aminobutanoate | HMDB0000112 |
| 4-hydroxy-2-oxoglutarate | HMDB0002070 |
| 4-methylpentanal | HMDB0001318 |
| 4-phosphooxy-L-threonine | HMDB0006802 |
| 5-aminolevulinate (methyl 5-aminolevulinate) | HMDB0015127 |
| 5'-deoxyadenosine | HMDB0001983 |
| acetyl-CoA | HMDB0001206 |
| acylglycerol | HMDBP08704 |
| acyl-sn-glycerol 3-phosphate | HMDBP04526 |
| arachidonate | HMDB0001043 |
| beta-amyrin | HMDB0249098 |
| β-D-galactopyranosyl-(1-4)- β -D-galactopyranosyl-(1-6)- β-D-galactopyranosyl-(1-3)- β -D-galactopyranose | N/A |
| betaine aldehyde | HMDB0001252 |
| butanoyl-CoA | HMDB0304279 |
| cardiolipin | HMDB0010226 |
| CDP-diacylglycerol | HMDB0006968 |
| coenzyme Q10 (Ubidecarenone) | HMDB01072 |
| coproporphyrinogen III | HMDB0001261 |
| dolichyl beta-D-mannosyl phosphate | HMDB0012218 |
| dolichyl phosphate | HMDB0006353 |
| ferricytochrome c | HMDB0012947 |
| FMNH2 | HMDB0001142 |
| glycerone phosphate | HMDB0001473 |
| isopentenyl diphosphate | HMDB0001120 |
| itaconate | HMDB0002092 |
| L-ascorbate | HMDB0000044 |
| L-carnitine | HMDB0000062 |
| L-galactono-1,4-lactone | HMDB0304401 |
| L-glutamate | HMDB0000148 |
| L-glutamate 5-semialdehyde | HMDB0002104 |
| L-methionine | HMDB0000696 |
| L-ornithine | HMDB0000214 |
| L-phenylalanine | HMDB0000159 |
| L-Threonine | HMDB0000167 |
| Margaroylglycine | HMDB0013246 |
| *O*-acetyl-(*R*)-carnitine | N/A |
| O-acetylcarnitine | HMDB0000201 |
| O-acetyl-L-serine | HMDB0003011 |
| *O*-hexadecanoyl-(*R*)-carnitine | HMDB0000222 |
| O-phospho-L-serine (Dexfosfoserine) | HMDB0000272 |
| oxidized rubredoxin | N/A |
| phosphatidate | N/A |
| phosphatidylethanolamine | HMDB0008828 |
| phosphatidylglycerol | HMDB0010570 |
| phosphatidyl-L-serine | HMDB0014291 |
| phosphocreatine | HMDB0001511 |
| plantacyanin | HMDB0303586 |
| polysulfide | N/A |
| pregnenolone | HMDB0000253 |
| prostaglandin H2 | HMDB0001381 |
| protoheme IX | HMDB0003178 |
| protoporphyrin IX | HMDB0000241 |
| protoporphyrinogen IX | HMDB0001097 |
| quinol | HMDB0002434 |
| reduced coenzyme Q10 | N/A |
| reduced rubredoxin | N/A |
| retinal | HMDB0001358 |
| retinoate | HMDB0001852 |
| sn-glycerol 3-phosphate (3-Phosphoglycerol) | HMDB0000126 |
| succinate semialdehyde | HMDB0001259 |
| succinyl-CoA | HMDB0001022 |
| sulfur- (sulfur carrier) | N/A |
| tetrahydrofolate (Tetrahydrofolic acid) | HMDB0001846 |
| Thymidine monophosphate | HMDB0001227 |
| trans-2(or 3)-enoyl-CoA | HMDB0003944 |
| trans-2,3-dehydroacyl-CoA | N/A |
| trimethylglycine | HMDB0000043 |
| ubiquinol | HMDB0001304 |
| ubiquinone | HMDB0001072 |
| UDP-glucose | HMDB0000286 |

* Human Metabolome Database identification number

After our initial search, 32,798 genes were identified by the National Center for Biotechnology Information's (NCBI) Gene database, 30,652 were categorized as eukaryotes genes, 1,348 bacterial, 521 of archaea and 277 as virus (**Supplementary Fig. S1**). The 30,652 genes categorized as only being present in eukaryotes were included while the rest were eliminated. After manually screening the eukaryote mitochondrial genes, the GeneID further reduced the list of mitochondrial genes from 30,652 to 2,682 which accounted for duplicates.


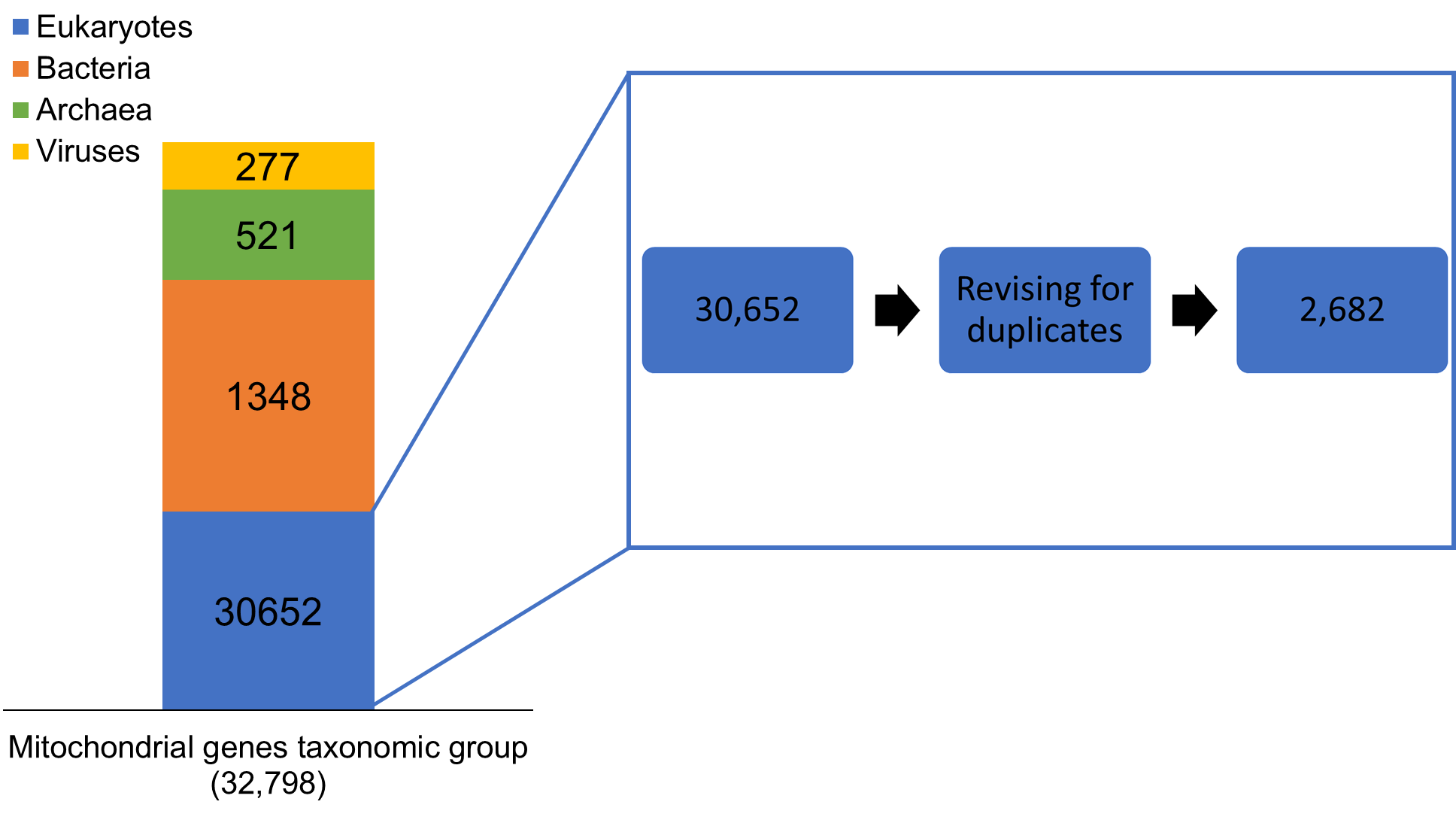


**Supplementary Figure S1:** Mitochondrial genes taxonomy distribution. Data reduction was achieved by further categorizing genes as eukaryote, bacterial, archaea, or virus, the eukaryote genes of interest were assessed for redundancy based on the gene description and duplicates were removed.

***In vivo* L-Carnitine challenge results**

L-carnitine was evaluated to assess the validity of our data mining strategy and its feasibility for clinical use as a marker of mitochondrial ADR using a mouse model. The results from these experiments are depicted in **Supplementary Fig. S2-S5.** These results show how CFZ treatment causes host perturbation of metabolic functions, observed in weight, food and water consumption, urine production and loss of skeletal muscle mass.


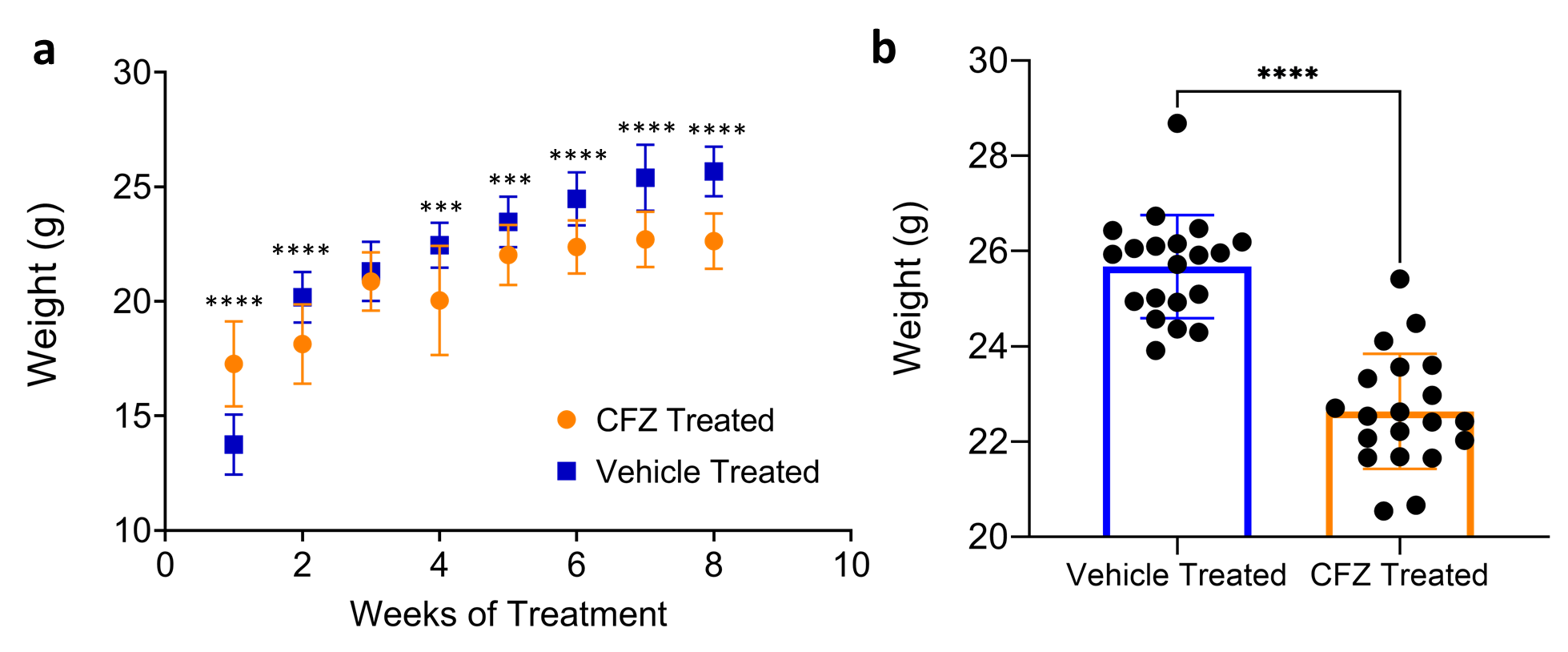


**Supplementary Figure S2.** Change in body weight over 8-weeks of clofazimine (CFZ) treatment. **a**) Weight (g) over the 8-week treatment of vehicle treated (blue) and CFZ treated (orange) mice. **b**) CFZ-treated mice weighed on average (SD) less (22.6+1.21g) than vehicle treated (25.7+1.08g) at the end of the 8-week treatment. (****p=<0.0001). Data are the mean (SD) of 20 mice/group.

Despite the differences in weight, both vehicle and CFZ treated mice consumed the same amount food per day (3.0 ± 0.28 g, p=0.58) (**Supplementary Fig. S3a**). However, CFZ treated mice consumed on average less water (2.6mL ±0.07) than vehicle treated (4.0mL ±0.60) mice (**p=0.007) (**Supplementary Fig. S3b**), which in turn led to CFZ treated mice having a lower average (SD) urine production over 24h (4.2mL ±1.05) than the vehicle treated (6.4mL ±0.60) mice (*p=0.022) (**Figure Supplementary Fig. S3c**).


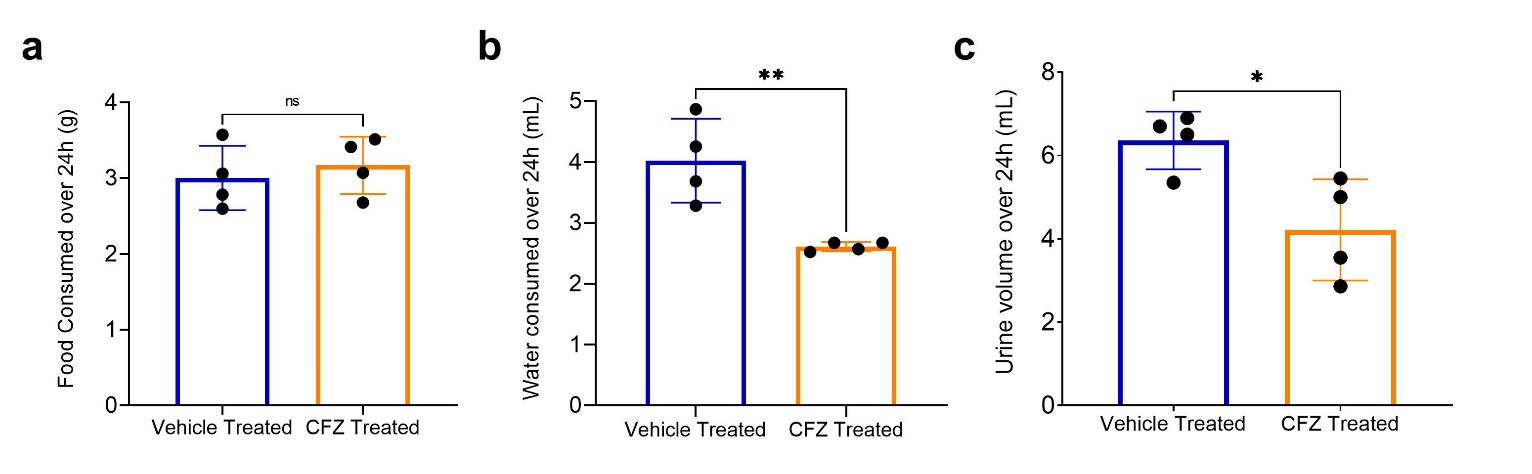


**Supplementary Figure S3**. Metabolic functions of vehicle treated (blue) and clofazimine (CFZ)-treated (orange) mice. **a**) In both groups, food consumption over 24h did not differ (3.0g ±0.42 vs 3.2±0.37 CFZ, p=0.58). **b**) CFZ treated mice consumed on average less water (2.6mL ± 0.07) than vehicle treated (4.0mL ±0.60) mice (**p=0.007) **c**) CFZ treated mice produced on average less urine (4.2mL ±1.05) than vehicle treated (6.4mL ±0.60) mice (*p=0.022). Data are the mean (SD) of 4 metabolic cages of 5 mice/group.

Another indication of metabolic alteration could be reflected in changes in skeletal muscle mass. In both the gastrocnemius and the quadricep muscle CFZ treated mice exhibited a decrease in muscle mass. For the gastrocnemius **(Supplementary Fig. S4a)** vehicle treated mice show an average mass of (153.3 ± 13.80g) when compared to CFZ treated mice (129.6 ± 15.18g, ****p=<0.0001). In the quadricep vehicle treated mice (163.1 ± 22.91g) while on average CFZ treated mice showed (147.1 ± 26.58g, *p=0.0484) **(Supplementary Fig. S4b).**


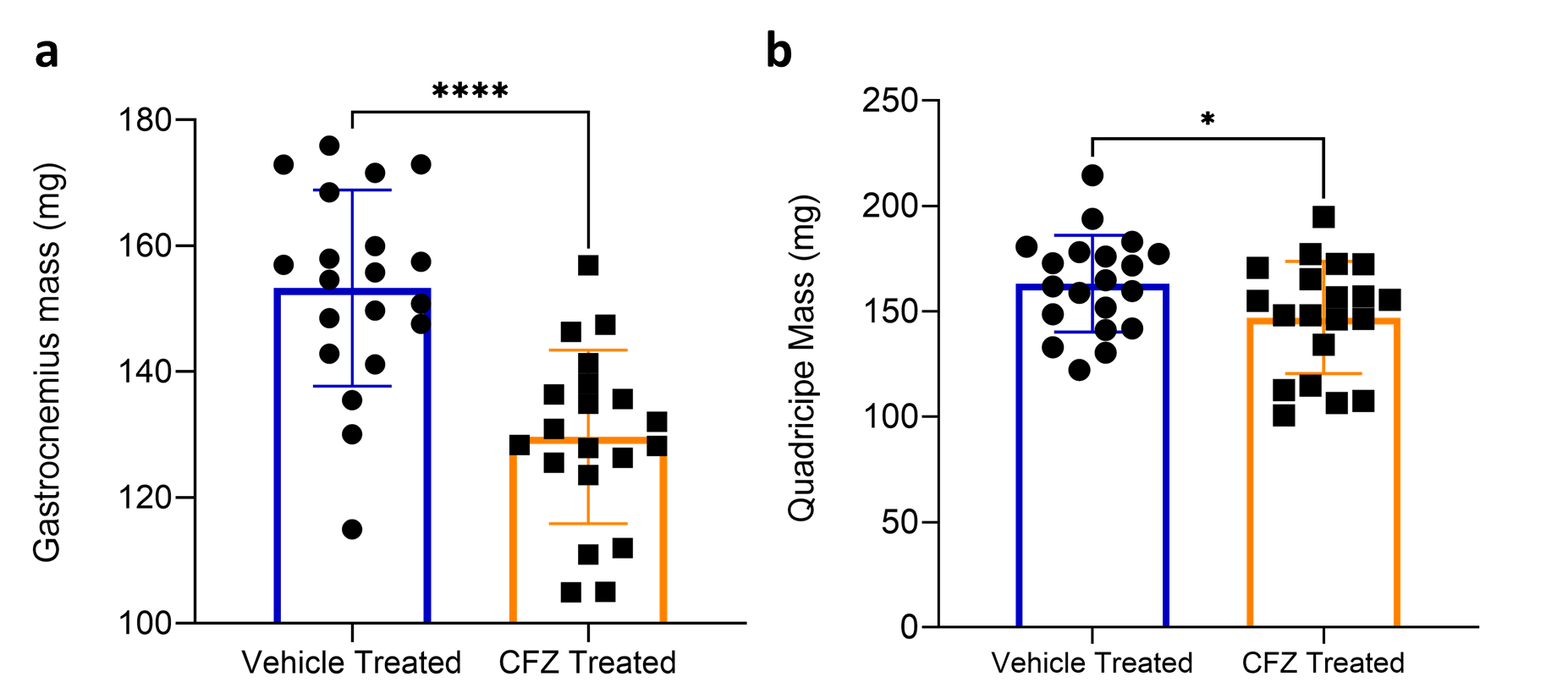


**Supplementary Figure S4**. Muscle mass of vehicle treated (blue) and clofazimine (CFZ)-treated (orange) mice. **a**) Gastrocnemius muscle mass in CFZ treated mice was lower (129.6 ± 15.18g), when compared to vehicle treated mice (153.3 ± 13.80g, ****p=<0.0001) **b)** CFZ treated mice exhibited lower quadricep muscle mass (147.1 ± 26.58g) when compared to the vehicle treated mice (163.1 ± 22.91g, *p=0.0484).

**Methods**

**Data base Screening and Process of Elimination Strategy**

*Rationale for Approach*

For the methodology, we used four different databases in sequential order for the initial portion of the analysis: The National Center for Biotechnology Information’s (NCBI) Gene database (<https://www.ncbi.nlm.nih.gov/gene>), The Braunschweig Enzyme Database (BRENDA) (<https://www.brenda-enzymes.org/>), the Kyoto Encyclopedia of Genes and Genomes (KEGG) database (<https://www.genome.jp/kegg/>), and the Universal Protein Resource (UNIPROT) database (<https://www.uniprot.org/>). These databases were accessed by an author’s (DP) computer using the web browser, Google Chrome (Version 116.0.5845.188 (Official Build) (64-bit)). The resulting metabolite candidates were then subjected to evaluation by three criteria that were assessed using: Drug Bank (<https://go.drugbank.com/>), the FDA Orange Book (<https://www.accessdata.fda.gov/scripts/cder/ob/index.cfm>), and the United States Pharmacopeia (USP; <https://www.usp.org/>). The rationale for the use of each database, the sequence of evaluation and the inclusion/exclusion criteria for each candidate are outlined in **Supplementary Table S2.** Database screening was performed between March-September/2021. Some screenshots were added at a later data as noted in **Supplementary Table S2**.

**Supplementary Table S2.** Database Screening and Process of Elimination Strategy and Rationale for Each Step

| Step | Rationale | Search Methodology, and Data Extraction and Handling |
| --- | --- | --- |
| The National Center for Biotechnology Information’s (NCBI) Gene Database (https://www.ncbi.nlm.nih.gov/) | The NCBI Gene database was selected as the starting point for our strategic database screening methodology because it integrates gene-specific information from multiple data sources which includes, NCBI Reference Sequence (RefSeq), organism databases and Gene Ontology. This allowed access to many different types of gene-specific data connected to the record. Mitochondrial genetic information has been previously used to establish causes of diseases, for example associations have been observed between mitochondrial haplogroups, a population who shares similar mtDNA sequence changes or polymorphism^1^, and predisposition to Parkinson's disease^2^, multiple sclerosis^3^, Leber hereditary optic neuropathy^4^, type 1 and type 2 diabetes, cardiomyopathies and stroke-like episodes^5^. The NCBI Gene database also collects taxonomic group information from the NCBI Taxonomy database^6^. It was important to make this distinction because the focus of the work was on mitochondrial genes and mitochondria are known to be present only in eukaryotic cells. | To identify mitochondria-related genes, on the NCBI website, “gene” was selected in the “all databases” drop down menu. The word “mitochondria” was used as the initial search word since the focus of our investigation was mitochondria related metabolites (see screenshot below). Only genes listed in the results table were considered further. Each of the identified mitochondria genes were categorized based on taxonomic domain, (eukaryotes, bacteria, viruses, and archaea), using the “results by taxon” filter (see screenshot below); genes exclusively present in eukaryotes were identified and viewed as a “tree” (rather than list). These gene names were copied to an excel spreadsheet and evaluated for any duplicates (the same gene present in different organisms) and these were removed from the dataset.  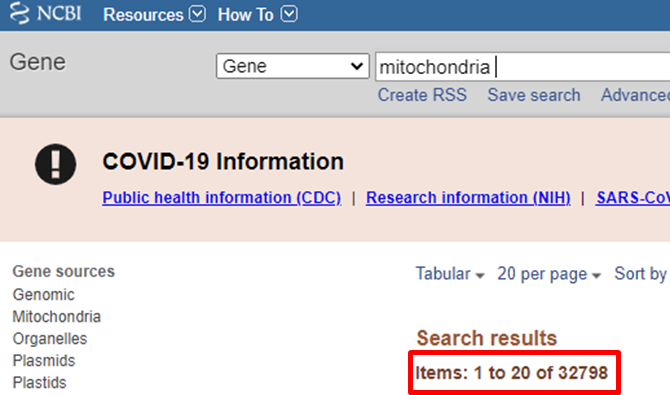  *Screenshot from NCBI website showing results from “mitochondria” gene search (website accessed June/2021).*  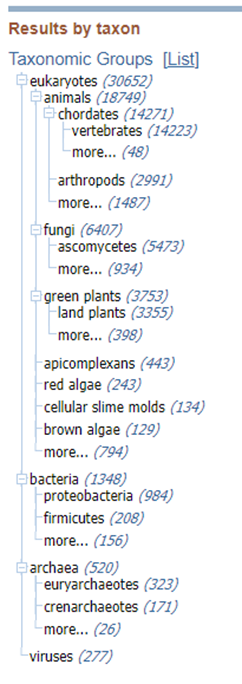  *Screenshot from NCBI website “results by taxon” filter (website accessed June/2021).* |
| BRENDA Database: Substrates and Products of Eukaryote Mitochondrial Genes (https://www.brenda-enzymes.org/) | BRENDA is a comprehensive relational database on functional and molecular information of enzymes, based on the primary literature^7^. BRENDA is an important tool for biochemical and medical research because it covers information on properties of all classified enzymes, including data on substrates/products, structure, and stability. BRENDA was used in the second step of the methodology to search for the enzymatic information of the identified mitochondrial genes and to identify the substrate and products of each of the reactions. This step was taken to eliminate any protein structural components and focus on the substrate and products (e.g., metabolites). | From the dataset curated from the NCBI Gene Database, the gene name was searched in BRENDA (“enzyme, ligand”) to identify associated enzymes. For each gene, this resulted in a list of enzymes and respective enzyme commission numbers (EC number), a numerical classification scheme for enzymes. Each EC number is hyperlinked to a general information page about the enzyme (see screenshot below). From this page, the substrate(s) and product(s) of each reaction were identified and combined in a spreadsheet to allow for the elimination of duplicates (e.g., compounds that participated in multiple reactions as either the substrate or product).  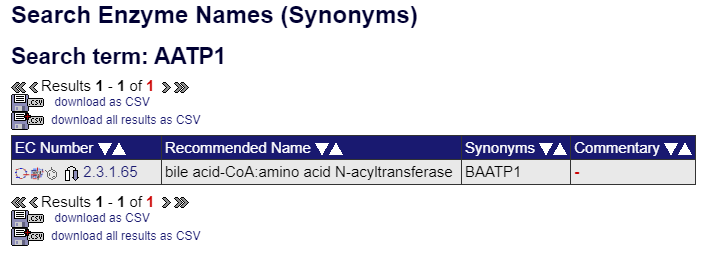  *Screenshot from BRENDDA website showing the enzyme linked to the mitochondrial gene AATP1 (website accessed October/2023).* |
| KEGG Database: Separations of Small Compounds and Structural Components (https://www.genome.jp/kegg/) | KEGG was used as the third step to categorize the identified substrates and products as small compounds using a molecular weight (MW) cutoff of ≤ 1000 daltons. Small compounds were of interest since the inner mitochondrial membrane favors uncharged, low molecular weight and lipophilic substances. | The compounds (substrates or products) identified from the previous step were evaluated using the KEGG database. From the KEGG home page, the KEGG compound database was accessed, and the name of each compound was entered into the search field. The MW (exact mass; see screen shot below) of each compound was recorded in Excel file. Those with a MW >1000 Daltons were removed from the data set.  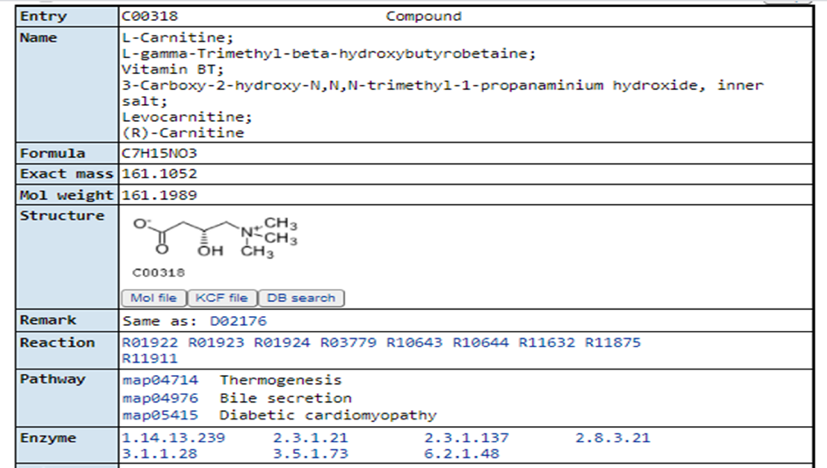  *Screenshot of L-carnitine information from KEGG website (KEGG compound) (website accessed October/2023).* |
| UNIProt Database: Specific Gene Location (https://www.uniprot.org/) | The last step in the workflow was to verify that the transformation of the candidate compounds was exclusively mediated in mitochondria. For this, the UNIPROT database was used. This database also provides a resource for protein sequences and functional information. | To confirm human genes and the subcellular location of the near final list of compounds as mitochondrial, each gene name was entered into the UNIPROT search tab. From the resulting table, human genes were selected, and the “subcellular location” feature was used to provide visual confirmation (see screenshots below).  *Example: Searching for the Gene “AIFM1”*  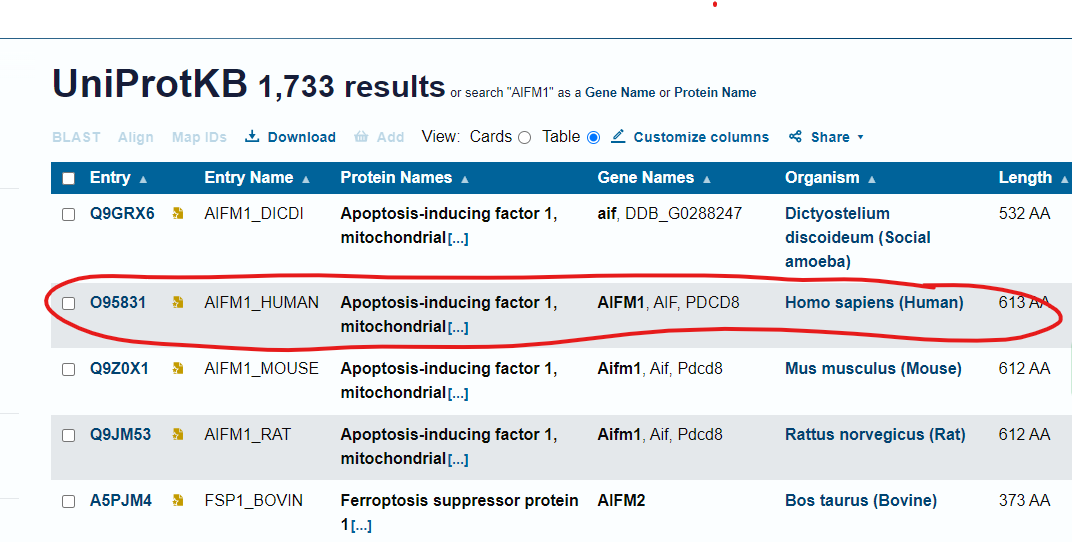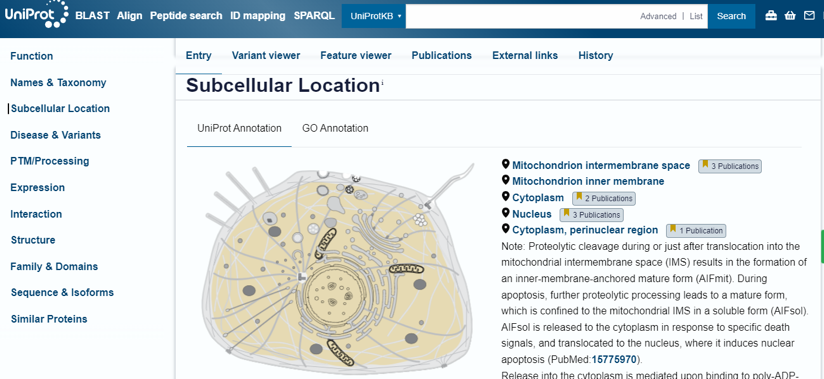*This gene (and its associated metabolites) would be eliminated because it is not exclusively present in mitochondria (website accessed October/2023).* |
| Application of final criteria: Physiochemical and pharmacokinetic properties of selected compounds | The final compound candidates were evaluated for clinical applicability using the following criteria: 1) availability of peer-reviewed literature documenting physiologic blood concentrations and pharmacokinetic data in both mice and humans, 2) an FDA approved formulation for intravenous use and 3) a commercially available enzyme-linked immunosorbent assay or published LC/MS analysis protocol for quantification in blood. The databases used for the application of these criteria were: Drug Bank (<https://go.drugbank.com/>), the FDA Orange Book (<https://www.accessdata.fda.gov/scripts/cder/ob/index.cfm>) and the US Pharmacopeia (<https://www.usp.org/>). We needed this information to be able to perceive and measure any changes of the compound’s levels during the challenge test. We needed the compound to be available as an FDA approved formulation, so it could be safely used and in compliance with animal use and care guidelines. Even though there are many available assays and LC/MS protocols can be developed, having a commercially available assay or an established LC/MS protocol for the selected compound would also facilitate the “challenge test”. |  |

Literature search strategy and evaluation criteria

A comprehensive literature evaluation of published research studies relating the three finalist compounds to ADR’s, mitochondrial function, and information about animal and human physiological blood concentrations (**Supplementary Table S3**). Each database was accessed via its website from an author’s (DP) computer (May-September 2021) using the web browser, Google Chrome (Version 116.0.5845.188 (Official Build) (64-bit))

**Supplementary Table S3.** Literature evaluation criteria for three finalist compounds

| Compound | trimethylglycine | L-carnitine | quinol |
| --- | --- | --- | --- |
| Databases searched between 05-2021 and 09-2021. | PubMed (<https://pubmed.ncbi.nlm.nih.gov/>)  Science Direct (<https://www.sciencedirect.com/>)  Google Scholar (<https://scholar.google.com/>) | | |
| Search Terms | “Trimethylglycine & mitochondria”, “trimethylglycine” & adverse drug reaction”, “trimethylglycine & biomarker”, “trimethylglycine & pharmacokinetics”, “beatine & mitochondria”, “beatine” & adverse drug reaction”, “beatine & biomarker” “beatine & pharmacokinetics” | “L-carnitine & mitochondria”, “L-carnitine” & adverse drug reaction”, “L-carnitine & biomarker” “L-carnitine & pharmacokinetics” | “quinol & mitochondria”, “quinol” & adverse drug reaction”, “quinol & biomarker” “quinol & pharmacokinetics”, “hydroquinone & mitochondria”, “hydroquinone” & adverse drug reaction”, “hydroquinone & biomarker” “hydroquinone & pharmacokinetics” |
| Article Type | Research Articles, Reviews, Systematic reviews, Clinical Trials, Case reports, Technical Reports | | |
| Species Included | Human, mice | | |
| Language | English | | |
| Year range | 1980- present | | |

**LC/MS Assay for Measurement of L-carnitine and Acetylcarnitine**

LC Conditions

L-carnitine and acetylcarnitine concentrations (ng/ml) were determined by the LC–MS/MS method developed and validated for this study. The LC–MS/MS method consisted of a Shimadzu LC-20AD HPLC system (Kyoto, Japan), and chromatographic separation of the tested compound was achieved using an Agilent Poroshell 120 EC-C18 column (3.0 x 100 mm, 2.7 µm) at 25 °C. Five microliters of the supernatant was injected. The flow rate of gradient elution was 0.35 ml/min with mobile phase A (10mM ammonium formate and 0.1% formic acid in purified deionized water) and mobile phase B (0.1% formic acid in acetonitrile). An Sciex QTOF X500R mass spectrometer equipped with an electrospray ionization source (ABI-Sciex, Toronto, Canada) in the positive-ion high resolution multiple reaction monitoring (HRMRM) mode was used for detection. Protonated molecular ions and the respective ion products were monitored at the transitions of m/z 162.11 > 103.0364 for L-carnitine, 204.12 > 85.0250 for acetylcarnitine, 166.14 > 103.0380 for L-carnitine-13C, D3 and 207.14 > 85.0255 for acetylcarnitine-D3. We adjusted the instrument settings to maximize analytical sensitivity and specificity of detection. Data were processed with software SCIEX OS (version 2.1.6).

LC–MS/MS analysis was performed by using an X500R QTOF (SCIEX). Components were separated on a 3.0 x 100mm EC-C18 column with 2.7μm particle size (Agilent). The mobile phase delivered at 0.35 mL/min was a mixture of (A) 10mM Ammonium formate and 0.1% formic acid in purified deionized water and (B) 0.1% formic acid in acetonitrile, using the following gradient elution: 0.01-1.50 min (1%B), 3.00-4.50 min (95%B), and 4.60-6.60 min (1%B).

**References:**

1. Chinnery, P. F. & Hudson, G. Mitochondrial genetics. *Br. Med. Bull.* **106**, 135–159 (2013).

2. Van Der Walt, J. M. *et al.* Mitochondrial polymorphisms significantly reduce the risk of Parkinson disease. *Am. J. Hum. Genet.* **72**, 804–811 (2003).

3. Yu, X. *et al.* mtDNA nt13708A variant increases the risk of multiple sclerosis. *PLoS One* **3**, 1–7 (2008).

4. Hudson, G. *et al.* Clinical expression of leber hereditary optic neuropathy is affected by the mitochondrial DNA-haplogroup background. *Am. J. Hum. Genet.* **81**, 228–233 (2007).

5. Chinnery, P. F. & Gomez-Duran, A. Oldies but goldies mtDNA population variants and neurodegenerative diseases. *Front. Neurosci.* **12**, 1–11 (2018).

6. Federhen, S. The NCBI Taxonomy database. *Nucleic Acids Res.* **40**, 136–143 (2012).

7. Schomburg, I., Chang, A. & Schomburg, D. BRENDA, enzyme data and metabolic information. *Nucleic Acids Res.* **30**, 47–49 (2002).
